# Career perspective

**DOI:** 10.1038/s44323-024-00004-2

**Published:** 2024-08-01

**Authors:** Sato Honma

**Affiliations:** Center for Sleep and Circadian Rhythm Disorders, Sapporo Hanazono Hospital, 1-30, South 15, West 15, Chuo-ku, Sapporo, 064-0915 Japan

**Keywords:** Neuroscience, Diseases

## All experiences matter as a scientist

Here are the four important insights from my life: the challenge of working in a new field is more thrilling than routine jobs; failure offers career growth; the true value of seemingly insignificant experiences become evident only when they are called into use later in life; and life is long and full of surprises.

My career as a scientist began in 1972 when I joined the Department of Physiology, Hokkaido University Graduate School of Medicine, Japan, as a Ph.D student after medical school. My Ph.D research was on sex differences in the circadian rhythms of hypothalamic–pituitary–adrenal (HPA) axis^1^. In 1970s, cutting-edge research in neuroendocrinology was advancing, and several novel neuropeptides were discovered successively. The 1977 Nobel Prize in Physiology and Medicine was awarded to Dr. R. Guillemin and Dr. A. Schally for their research on peptide hormone production in the brain. My supervisor Dr. Hiroshige had developed a corticotrophin-releasing hormone (CRH) bioassay in 1968. CRH remained unidentified until Vale et al. characterized it in 1981. Therefore, until then, Dr. Hiroshige’s bioassay was one of the limited tools to analyze CRH activity. HPA axis exhibits a marked circadian rhythm. Thus, my research began by studying biological rhythms.

After Ph.D, I struggled to find work as a researcher. Japan did not have a postdoctoral system then, and applying for research positions was extremely competitive. Despite the tenure-system, one had to wait for a long time until someone quit. Being married and a mother of a 3-month-old daughter, it seemed almost impossible to find a position. In addition, until the 1990s, female medical students were commonly regarded as a waste of tax money since many of them could not continue career due to gender bias and a lack of support systems. Therefore, I worked as a resident pediatrician at Hokkaido University Hospital. I skipped many clinical-training sessions held in the evening to fetch my daughter from preschool. Despite mothers not being welcomed as doctors, I found the work stimulating. I realized the true value of this experience only after working as a professor.

## 5 years and 1 day

It took me 5 years and 1 day after completing my Ph.D to be hired as an assistant professor at Hokkaido University; this was the exact duration for which the repayment for the graduate scholarship loan I had taken was suspended. Two assistant professors quitted simultaneously, forcing the Department of Physiology to hire someone immediately. And I was on the waiting list for 5 years. Until then, I worked as a resident pediatrician and volunteer research staff at the Neurochemistry Department, Max Planck Institute, Göttingen, Germany, and at the Anatomy Department, Hokkaido University Graduate School of Medicine. Therefore, my advice to young women researchers is to follow your dreams, persevere, always stay positive, and never hesitate to ask for help.

## Chance favors only the prepared mind ~Louis Pasteur

In the Neurochemistry Department, Max Planck Institute for Biophysical Chemistry, Göttingen, research on synaptic mechanisms using synaptosome obtained from the electric organ of *Torpedo marorata* (marbled electric ray) was ongoing. The organ is of muscle origin and spindle-shaped in fetal *Torpedo* that later develops into a piled coin-like structure in adults. Transmission electron microscope images suggested that the entire electric organ surface is covered with presynaptic terminals. My assignment was to image what does it look like using a scanning electron microscope. After trying for 5 months to image its surface and follow the nerve terminals, one day, in a weak collagenase-treated specimen, I finally observed the cleft of the piled coin structure and located the nerve terminals; they branched and flattened, covering the entire cell surface. A few days later, I re-examined the samples once I thought I failed and found the nerve terminals were there. Lacking a prepared mind, I could not see my target which was right in front of my eyes. This experience reminds me of what Nobel laureate Albert Szent-Györgyi (1962) said: “Discovery is to see what everybody has seen, and think what nobody has thought.”

## Believe in yourself

I was 33 years old when I became an assistant professor. Until then, I could not apply for a grant, making it a late start. Maternal entrainment of fetal and newborn animals was the first subject I undertook as an assistant professor. The experimental design involved preparing light–dark (LD)- and DL-entrained mothers and exchanging mother rats every 12-h to expose pups to either LL or DD. However, this was difficult due to my daughter’s school timings. Instead, I proposed making a suprachiasmatic nucleus (SCN) lesion in the pregnant mother, eliminating maternal rhythms. Other members objected as there was a risk of the SCN-lesioned mother neither retaining the pregnancy nor nursing the pups. However, I insisted based on the finding that if the lesion was precisely restricted in SCN, the mother could deliver and nurse pups. My necessity became the mother of a new finding: prenatal start of circadian oscillation and maternal entrainment of fetal clock^2^. Do not hesitate to do what you believe in as you learn something even when you fail, and nothing is more regrettable than not trying.

Having a “prepared mind” also helped to find an SCN-independent, methamphetamine-induced rhythm. After observing our preliminary behavior activity data under chronic methamphetamine treatment, Dr. A Borbély, who was visiting, suggested to exclude the possibility that the rhythm was drug-induced hyperlocomotion followed by rest. We used the retired SCN-lesioned female rats from entrainment experiments to immediately test the hypothesis that the rhythm is induced by an extra-SCN oscillating system^3^.

## We need mentors, collaborators, and good competitors

Nowadays, an academic mentoring system is available for any researcher in need. But good mentors and advisors in the research career are not always assigned by your institution. I am thankful for Dr. Jürgen Aschoff who always encouraged young chronobiologists including me. I am thankful for my collaborators who accompanied me through my career. It is impossible to experiment alone, particularly considering research advancement speed and entailing complexities. Having specialist collaborators is vital for a successful career in science. Such collaborations led to a breakthrough in my career^4,5^. Most importantly, a good competitor is sometimes more helpful than collaborators; Competitors stimulate thought and make life colorful. I am thankful for all wonderful mentors, collaborators, competitors, and, especially, for my family.

## Data Availability

No datasets were generated or analyzed during the current study.
